# An Inclusive View of the Disability of Secondary School Students

**DOI:** 10.3390/ijerph17238922

**Published:** 2020-11-30

**Authors:** Cristina Méndez-Aguado, Rubén Trigueros, José Manuel Aguilar-Parra, Noelia Navarro-Gómez, Mª del Pilar Díaz-López, Juan M. Fernández-Campoy, Juan Gázquez-Hernández, José Carrión

**Affiliations:** 1 Hum-878 Research Team, Health Research Centre, Department of Psychology, University of Almería, 04120 Almería, Spain; cma824@inlumine.ual.es (C.M.-A.); jfc105@ual.es (J.M.F.-C.); gazquezhernandez@gmail.com (J.G.-H.); 2 Hum-760 Research Team, Health Research Centre, Department of Psychology, University of Almería, 04120 Almería, Spain; nng777@ual.es; 3 Hum-498 Research Team, Health Research Centre, Department of Nursing Science, Physiotherapy and Medicine, University of Almería, 04120 Almería, Spain; dlm477@ual.es; 4 Department of Education, University of Almería, 04120 Almería, Spain

**Keywords:** emotional intelligence, psychological flexibility, prosocial behaviours, inclusive behaviours, inclusion

## Abstract

Achieving the educational inclusion of students with special educational needs (SEN) is one of the significant challenges of the current Spanish educational system. This is a group of students with a high rate of bullying that leads to academic failure, as well as significant psychological and social consequences. Despite the fact that the behaviours and psychological characteristics of their peers seem to influence the degree of inclusion, there is no detail on this subject. Therefore, the aim of this paper is to determine the relationship between emotional intelligence, psychological flexibility, prosocial behaviour and inclusive behaviour. To carry out this study, a sample of 642 students between the ages of 12 and 19 years old participated and answered four questionnaires, one for each variable under study. The relationships established were extracted from different statistical analyses and a hypothesised predictive model. The results obtained revealed that emotional intelligence is positively related to psychological flexibility and prosocial behaviour and that these, in turn, are positively related to the development of inclusive behaviour. Therefore, the importance of considering the variables under study during the teaching–learning processes carried out in the classroom is highlighted.

## 1. Introduction

Achieving inclusive education is one of the main challenges faced by different education systems. Numerous studies have covered this issue, focusing on teachers themselves, considering both their personal characteristics and the teaching methodologies used [1]. In this way, the investigation into the influence of psychological and behavioural characteristics of students who share a classroom with special educational needs (SEN) students has been put on the back burner [2]. However, these characteristics are related to issues of great current relevance, such as school bullying [3,4]. This is a group that presents a greater incidence in this regard, corresponding to 80.3% of the total rate of bullying and predominantly to physical disabilities [5]. As a result, they are more vulnerable to school failure due to the psychological, social and educational conditions that it entails [6]. Therefore, it is necessary to study in depth the influence of these personal characteristics on the inclusion of students with SEN.

### 1.1. Emotional Intelligence

Based on studies related to multiple intelligences, Salovey and Mayer developed the concept of emotional intelligence in the 1990s [7], considering it as a capacity related to the identification, assessment, understanding and expression of one’s own emotions and whose regulation promotes positive personal development [7]. These aspects constitute the main axis of its model of emotional skills [8], thus considering this intelligence as a mental ability. In contrast, the models proposed by Goleman and Bar-On are of mixed type, since they take into consideration both the mental perspective and its combination with the personality traits [9]. Despite these similarities, both differ in the focus of the model. The model developed by Goleman is aimed at work success and constitutes the recognition and emotional control, self-motivation, recognition of other people’s emotions, favouring respect through empathy and control of social skills [10]. Regarding the Bar-On proposal, its social aspect stands out and it is composed of five factors known as intrapersonal and interpersonal skills, adaptability, stress management and general mood [11].

Considering the study of emotional intelligence from an educational perspective, research has established its influence on the academic performance of students, regardless of age or subject [12,13]. Likewise, there is a positive correlation between the factors that make up emotional intelligence, including those most related to social skills [14,15]. However, although the models presented consist of factors such as adaptability and the development of social relations, related to psychological flexibility and prosocial behaviour, these are variables that are rarely considered from the point of view of inclusion. Existing studies have determined that there are factors of emotional intelligence, such as recognition and emotional expression, that need psychological flexibility [16], just as low emotional intelligence has been related to psychological inflexibility [17]. Likewise, the existence of a positive correlation between this intelligence and prosocial behaviour has been established, whether considered in a general way [18,19] or by delving into the components of emotional intelligence [20,21]. However, in both cases, the absence of previous studies carried out from the perspective of students without SEN and their repercussions on those who do have this type of need are noteworthy.

### 1.2. Psychological Flexibility

The ability to remain in the present and the situation that is taking place, by making modifications in behaviour that allow for greater adaptation, is called psychological flexibility [22], a concept opposed to psychological inflexibility, related to experiential avoidance and the consideration of thought as a reality and not as previous experience [23]. It is composed of six interdependent processes collected in the model of Hayes et al. [22], later called hexaflex [24], based on acceptance and commitment therapy. These authors, together with the contributions of Hayes [25], specified their processes, which allow for the recognition of internal events, the development of detachment from these events in order to favour bending, maintaining attention on the present situation while being aware of the thoughts, actions and emotions experienced and establishing the values that will subsequently influence the actions carried out. Applying this model to the field of inclusion, the development of its different components allows for awareness of prejudice by favouring detachment and promoting social relations guided by positive values [26,27,28]. So that it is a variable related to the reduction of discrimination and stigma developed towards vulnerable groups [27,29].

Psychological flexibility has been mostly studied in the area of health, noting its positive relationship with the reduction of addiction to substances such as tobacco [30] and with greater personal well-being and lower levels of stress [31], as well as the predominance of rigid thinking in the face of disorders such as [32]. However, there are also studies applied to the educational context, relating psychological flexibility with less procrastination [33] and psychological inflexibility with the emergence of racial prejudice [34]. However, no previous relationship has been established between psychological flexibility and improved social relations, so there is no empirical evidence on the influence it may have on the development of inclusive behaviours towards students with SEN.

### 1.3. Prosocial Behaviour

This type of behaviour includes those actions carried out for the benefit of others and without expecting something in return [35], as well as those actions that should not act as a detriment to the recipient [36]. Three main subcategories can be distinguished within pro-social behaviour. On the one hand, there are the actions aimed at helping the other so that it is a matter of contributing to the well-being of others. On the other hand, there is altruism whose purpose continues to be the benefit and welfare of the other but, in this case, one acts selflessly. Finally, we speak of cooperation when different people work together to achieve a common goal [37]. Its acquisition is influenced by various factors including cultural, personal and intrinsic, to the situation and the family and school context [38].

Studies in this regard show the positive relationship between prosocial behaviours and empathy [39]. In the same way, they show the relevance of different factors like the family context that acts positively when there is good communication between the members of the family [40] and/or the school context in which the cohesion of the class group influences the development of prosocial behaviours [41]. On the other hand, the study carried out by Pinto, Baines and Bakopoulou [42] determines that the development of contact between students with and without SEN favours the appearance of inclusive behaviours, so there could be a link between prosocial behaviours and this. However, there are no previous studies in which this relationship has been considered directly and from the perspective of students.

### 1.4. Inclusive Behaviour

In the educational field, students with a disability or chronic illness must face different barriers that compromise their participation and learning. Among them are negative attitudes, related to exclusion and discrimination based on the re-creation of negative stereotypes [43]. However, this is a barrier on which the school can act by promoting the reduction of prejudice through contact between its members [44], including the development of relationships between students with and without disabilities. In this regard, De Boer, Pijl and Minnaert [45] found that positive attitudes among non-disabled students promote the participation of those who do have a disability, a fact promoted by the positive correlation between attitudes and inclusive behaviour [46].

Inclusive behaviours have been contemplated mainly by looking at teachers and their own teaching strategies, determining that their attitude and knowledge about inclusion have a positive correlation [47], as well as that teachers’ skills and a positive classroom climate favour inclusion, while competitiveness and heterogeneous assessment hinder it [48]. Considering the perspective of the student body, although research is less abundant, it shows the coexistence of both positive and negative behaviours [49] and establishes that the the development of positive attitudes influences the promotion of inclusive behaviours [46]. Given the possibility of behaviour modification, the importance of developing programs aimed at promoting inclusive behaviours is highlighted, in which direct interaction between students should predominate [50,51], so that they act as a means of awareness and acceptance towards this group [52].

### 1.5. Objectives and Hypothesis

Although it is true that, at present, inclusion is one of the great educational challenges and is the focus of attention of numerous studies, these focus on aspects that are more centred on teachers and on learning methodologies and strategies, highlighting the absence of studies that go into the psychological and behavioural characteristics of students and establish relationships between them. In this sense, the main objective is to determine the relationship between emotional intelligence, psychological flexibility, prosocial behaviour and inclusive behaviour, establishing the following as the starting hypothesis for this research: (1) emotional intelligence will be positively related to psychological flexibility; (2) emotional intelligence will be positively related to prosocial behaviours; (3) psychological flexibility will positively influence inclusive behaviours; (4) prosocial behaviours will be positively related to the development of inclusive behaviours.

## 2. Method

### 2.1. Participants

The participants in the study were 642 secondary school students from the province of Almeria (Spain), aged between 12 and 19 years (M = 15.37; SD = 1.97). The selection of participants followed an incidental non-probabilistic sampling. The criterion for their participation was the delivery of informed consent, signed by their parents.

### 2.2. Measurements

Emotional intelligence. The Spanish version of Fernandez-Berrocal, Extremera and Ramos [53] of the Trait Meta Mood Scale 24 (TMMS-24) by Mayer, Caruso and Salovey [54] was used. This scale is composed of 24 items that are equally distributed among three factors: attention, clarity and repair. The instrument is scored on a Likert scale from 1 (no agreement) to 5 (full agreement).

Flexible thinking. The Acceptance and Action Questionnaire-Stigma by Levin, Luona, Lillis, Hayes and Vilardaga [27], validated in the Spanish context by Trigueros, Navarro-Gómez, Aguilar-Parra and Cangas [55], was used. This questionnaire consists of 21 items distributed between two factors: psychological flexibility, 11 items; and psychological inflexibility, 10 items. The subjects had to indicate their answer by means of a Likert scale from 1 (never true) to 7 (always true). It should be noted that the items on the psychological inflexibility subscale were rated backwards, so low scores on this subscale indicate high flexibility of thinking.

Prosocial behaviour. The factor with the same name, belonging to the questionnaire Prosocial and Antisocial Behavior in Sport Scale (PABSS) by Kavussanu and Boardley [56], validated and adapted to the Spanish educational context by Trigueros, Alias, Gallardo, García-Tascón and Aguilar-Parra [57], was used. The factor is made up of seven items. The students had to answer each one of the items through a Likert scale from 1 (totally disagree) to 7 (totally agree).

Index for inclusion of students. The Spanish version validated by Fernández-Archilla, Álvarez, Aguilar-Parra, Trigueros, Alonso-López and Echeita [58] was used. The questionnaire is made up of a total of 38 items distributed towards a single factor called attitude towards inclusion. The students had to answer by means of a Likert scale from 0 (disagree) to 2 (agree).

### 2.3. Procedure

Initially, the management team of several educational centres were contacted, informed of the objective of the study, and their collaboration was requested in order to carry out the study. Subsequently, the parents or legal guardians of the students were asked for written permission for their children to participate in the study, explaining the objective of the study in an information note. Once informed consent was obtained, the questionnaires were administered to the students. The students completed the questionnaires at the beginning of the class after recess, individually with their classmates and under the supervision of a member of the research group to answer any questions that might arise.

The study was approved by the bioethics committee of the University of Almeria (Ref.157/2019). In addition, all the ethical postulates established in the Helsinki Declaration and the American Psychology Associations were respected.

### 2.4. Data Analysis

The statistical analyses used were: the mean, standard deviation, Pearson’s correlations, reliability analysis with Cronbach’s alpha index and a structural equation model (SEM). AMOS v20 (IBM, Armonk, NY, USA) and SPSS v25 (IBM, Armonk, NY, USA) statistical packages were used.

For the SEM, a bootstrapping of 10,000 interactions was used together with the maximum likelihood procedure for the estimation of the model. The estimators were considered robust despite the lack of normality [59]. The established fit indices for the SEM were: χ2/df between 2.00 and 3.00; Tucker–Lewis index (TLI), comparative fit index (CFI), incremental fit index (IFI) above 0.95; RMSEA below 0.06 and the SRMR below 0.08.

## 3. Results

### 3.1. Preliminary Analysis

Table 1 shows the mean, standard deviation and bivariate Pearson correlations, showing a positive correlation between the variables of the study. The reliability analyses reflected a score above 0.80.

### 3.2. Structural Equation Model

In order to carry out the SEM, it was necessary to reduce the number of latent variables by at least two indicators per factor due to the complexity of the model [60]. Thus, flexible thinking included two indicators (psychological flexibility and psychological inflexibility [55]). On the other hand, with regard to emotional intelligence, the items were specified in three indicators (attention, clarity and reparation [53]). However, for prosocial behaviour, it was necessary to separate the seven items into two indicators and the inclusion index was necessary to separate the 38 items into six indicators, all in order to identify the model, as suggested by McDonald and Ho [60].

**Figure 1:**
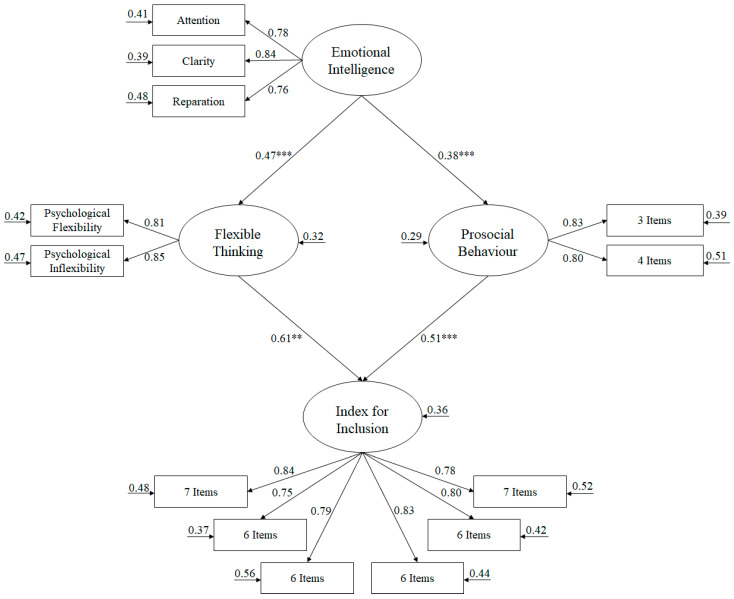
Structural equation model, showing all parameters and being statistically significant. Note: *** *p* < 0.001; ** *p* < 0.01.

The SEM adjustment rates (Figure 1), showed the following scores: χ^2^ (61, *N* = 642) = 166.82, χ^2^/df = 2.73, *p* < 0.001, IFI = 0.98, TLI = 0.98, IFC = 0.98, RMSEA = 0.052 (IC 90% = 0.043–0.061) SRMR = 0.039. The contribution between each of the factors was made through standardised regression weights.

The relationships obtained between the different factors comprising the model are described as follows:

(a) Emotional intelligence was positively related to flexible thinking (β = 0.47, *p* < 0.001) and prosocial behaviour (β = 0.38, *p* < 0.001).
(b) Flexible thinking was positively related to index for inclusion (β = 0.61, *p* < 0.001).
(c) Prosocial behaviour was positively related to index for inclusion (β = 0.51, *p* < 0.001).

## 4. Discussion

The main objective of this study has been to determine the relationship between emotional intelligence and psychological flexibility and prosocial behaviour and, at the same time, between the latter two variables and inclusive behaviour. These variables have been considered in several studies applied to different fields, among which the educational one stands out. However, we have no evidence of previous research studying the relationship between them and how emotional intelligence, prosocial behaviour and psychological flexibility influence the development of inclusive behaviour, i.e., how these variables, studied through students without SEN, affect the inclusion of their peers with SEN.

The results obtained in this study reveal the existence of a positive relationship between emotional intelligence and psychological flexibility, thus confirming the first hypothesis considered. Although these results cannot be contrasted with previous studies due to their absence, there are contributions in line with them. Research such as that of Bar-On [11] and Salovey et al. [8] determine that emotional intelligence leads to a greater capacity for recognition and emotional management, and therefore it can be deduced that these are people who face emotions instead of avoiding them, a characteristic of psychological flexibility. In the same way, emotional intelligence influences mindfulness and its effects, considering it as the ability to remain in the present without being influenced by prejudice [61]. Taking into account that mindfulness is related to psychological flexibility in a positive way [62], a similar relationship in this study seems to be established.

On the other hand, the second hypothesis can also be confirmed, since a positive relationship has been obtained between emotional intelligence and prosocial behaviour, a result in the same sense as those obtained by Poulou [63]. Likewise, other authors such as Martí-Vilar, Serrano-Pastor and Sala [9] determined a similar relationship, concluding that the mixed model proposed by Bar-On [11] is the greatest predictor of this type of behaviour. More specifically, Ruvalcaba-Romero, Gallegos-Guajardo and Fuerte [44] showed that social–emotional competencies such as adaptability or interpersonal competence predict the appearance of prosocial behaviours. Likewise, not only is emotional intelligence related to these behaviours, but they also influence positive family experiences, determining their future development [64]. In this way, the results of this study can be explained by the fact that emotional and social skills allow us to understand and control our own emotions, to be aware of other people’s needs and to maintain positive relationships, so that the development of prosocial behaviour is made possible [65].

As for the relationship established in psychological flexibility and inclusive behaviours, this has also proved to be positive, so that the third hypothesis is confirmed. Although the absence of research on this subject makes it difficult to compare these results, studies such as that of Brassell et al. [66] relate psychological flexibility with good family bonding, thus reducing conflict [67] by reducing negative parental thoughts and reactivity [68]. If these conclusions are applied to the field of educational inclusion, psychological flexibility would intervene in the improvement of the relationships that occur between students with and without SEN and would favour the emergence of inclusive behaviours.

With regard to the fourth hypothesis, in which a positive relationship between pro-social and inclusive behaviours was determined, it should be noted that it has been confirmed. This result has shown in agreement with Nelissen, Hülsheger, van Ruitenbeek and Zijlstra [69], in whose study it was determined that a prosocial motivation in employees positively influenced the development of inclusive behaviours towards people with disabilities. This relationship may be due to the similarities between both variables. However, while prosocial behaviours are related to support and empathy [70], those of an inclusive nature go beyond this, to allow people with disabilities to have the same opportunities and be considered as equals.

However, this study has a number of limitations. Firstly, as it is transversal research, it does not allow the extrapolation of cause–effect relationships in such a way that we have tried to establish relationships on the basis of possibility and not causality. Secondly, the selection of the sample was non-probabilistic and incidental. Likewise, future research should analyse these variables in samples comprising parents and teachers, in order to determine how they contribute to the inclusion of students with SEN.

In this way, the present study shows that we are all different, with strengths and weaknesses, with unique personalities and with different talents and interests. Through the diversity of an educational centre, the teacher should try to see the positive side of heterogeneity, the possibilities of learning and the values that can be extracted from the peculiarities of their students, promoting cooperative and dialogical activities. In this way, the students interact daily with each other, trying to work as a team to solve the different activities and tasks, and learning from each other, in order to break down barriers.

## 5. Conclusions

According to the results obtained, the research carried out has allowed us to determine the relationship between emotional intelligence, psychological flexibility, prosocial behaviour and inclusive behaviour, highlighting, therefore, the importance of attending to them when they are presented as factors that influence the educational inclusion of students with SEN. In this way, it is intended to make teachers aware of this fact, providing empirical evidence that the inclusion of students with SEN is not only achieved by involving teachers but also by considering the role played by their peers.

## Figures and Tables

**Table 1:** Descriptive statistics and correlations between all variables.

Factors	*M*	*SD*	Range	α	1	2	3	4
1. Emotional Intelligence	3.79	0.68	1–5	0.83		0.41 ***	0.53 ***	0.29 ***
2. Flexible Thinking	5.51	0.89	1–7	0.80			0.37 **	0.51 ***
3. Prosocial Behaviour	5.78	0.96	1–7	0.86				0.68 ***
4. Index of Inclusion	1.76	0.65	0–2	0.82				

Note: *** *p*< 0.001; ** *p* < 0.01.
